# Married very young adolescent girls in Niger at greatest risk of lifetime male partner reproductive coercion and sexual violence

**DOI:** 10.1371/journal.pone.0231392

**Published:** 2020-04-13

**Authors:** Stephanie M. DeLong, Mohamad I. Brooks, Sani Aliou, Rebecka Lundgren, Caitlin Corneliess, Nicole E. Johns, Sneha Challa, Nicole Carter, Giovanna Lauro, Jay G. Silverman

**Affiliations:** 1 University of California, San Diego, La Jolla, California, United States of America; 2 Pathfinder International, Watertown, Massachusetts, United States of America; 3 Pathfinder International, Niamey, Niger; 4 Promundo–United States, Washington, DC, United States of America; University of Westminster, UNITED KINGDOM

## Abstract

**Objective:**

The purpose of this analysis was to compare and contrast reproductive health (RH), gender equity attitudes, and intimate partner violence (IPV) among married very young adolescent (VYA) girls with married older adolescent girls and young women (AGYW) in rural Niger given limited literature on the topic.

**Methods:**

We conducted an exploratory analysis of baseline data from the Reaching Married Adolescents Trial in Dosso region, Niger. We report counts and percents, by age group (13–14 years (VYA), 15–16 years, 17–19 years), of AGYW’s self-efficacy to use family planning (FP), accurate knowledge of FP, current use of modern FP, and unintended last pregnancy (UIP); lifetime reproductive coercion (RC), physical IPV, and sexual IPV; and gender equity attitudes. We also assess whether percents differ between VYA and older groups using Pearson’s Chi-Square and Fisher’s exact p-values. Results are stratified by parity. Finally, we use logistic regression to consider associations.

**Results:**

There were 49 VYA, 248 girls aged 15–16, and 775 AGYW aged 17–19 in our sample (n = 1072). Accurate knowledge of FP, self-efficacy to use FP, current use of modern FP, and UIP increased with age; all percents between VYA and AGYW 17–19 were marginally or statistically significantly different. We also saw VYA report higher lifetime RC and sexual IPV versus older groups, with sexual IPV statistically different between VYA and girls 17–19. Parous VYA reported a significantly higher percent of lifetime RC versus older AGYW. Among 17–19 year-olds, odds of current use of FP were higher among AGYW who reported physical IPV, and odds of UIP were higher among those reporting more gender equitable attitudes, both adjusted for parity.

**Conclusions:**

We observed differences in RH, RC, and sexual IPV among married VYA and older AGYW in rural Niger. VYA should be prioritized in research to confirm and further understand their RH needs.

## Introduction

Niger is a mainly rural country of approximately 23 million people (2019)[1], with problems of food scarcity[2] and lack of economic opportunity[3]. Most Nigerien girls marry during adolescence (76% under 18 years, 28% under 15 years[4]) and bear children during this same period, a practice that may result in poor infant[5–7] and maternal health[6, 8] outcomes, as some studies in other nations have suggested. Adolescent modern family planning (FP) use is low (7%)[9].

Men’s greater power over FP decisions as compared to that of their young wives may, in part, be responsible for the low prevalence of modern FP use. Qualitative data from Maradi, Niger, suggests a husband’s permission, or fear of not getting that permission, limits some women from starting FP[10]. This evidence is also supported by qualitative data from Niamey, Tahoma, and Zinder, Niger, that suggests husbands may control or advise women about their FP use[11]. Nigerien men may be reluctant to allow their wives to use family planning because they want more children than women[11] or out of concern that the use of family planning would enable their wives to engage in extra-marital sex without the fear of pregnancy while husbands migrate for work (Personal communication, Dr. Sani Aliou, October 11, 2019). Wives may be at risk of reproductive coercion (coerced pregnancy, contraceptive sabotage; RC) when their views diverge from those of their husbands[12]. These same women may be at greater risk of experiencing intimate partner violence (IPV) given its association with RC[13].

### Married very young adolescent girls

Such relational power differentials and discordant fertility desires between husband and wife may be especially harmful for the married very young adolescent (VYA) girl (defined as ages 10–14 years[14]) in Niger. This is because of the physical, emotional, and social status of a very young adolescent as compared to an older adolescent (defined as ages 15–19[15]; we will further split this group into ages 15–16, representing middle adolescence, and 17–19, representing late adolescence, for analysis purposes). Very young adolescent girls are less developed physically than older adolescents[16], given the former are transitioning out of childhood. They are characterized by their small, growing body frames[17], and as a result, may be more likely to be injured than older adolescents during sex and childbirth because of their still small sizes. Very young adolescence is also a time normally characterized by puberty onset[17]. Dependent on age of marriage, however, menarche may not have occurred for VYA girls in Niger (Personal communication, Dr. Sani Aliou, October 11, 2019), making them unable to bear children in comparison to most girls in older adolescence. This has the potential to lead to emotional and social harm for the VYA girls in their marriages and communities.

The brains of VYA girls are also different developmentally as compared to older adolescents and may influence how VYA respond in situations. For example, grey matter density in the frontal cortex (responsible for cognition[18]) decreases from childhood[19] or around age 11[20, 21] across adolescence, with a continued increase of white matter during adolescence that improves the speed at which the brain relays and uses information[19–21]. This means that as girls are aging, their cognitive abilities (e.g., memory, ability to decide on socially-acceptable behavioral responses[18]) are enhanced. Steinberg et al also found that adolescents aged 13 and younger were less future-focused as compared to those aged 16 and older[22]. Further, McGivern et al have found that facial emotional processing time, but not accuracy, slows around puberty (age 11 for girls in this study), and then returns to a higher speed by age 15[23]. Overall, the less developed state of the VYA brain has the potential to impact Nigerien VYA girls’ knowing how or when to best advocate for their own sexual or reproductive desires, especially given VYA focus on the immediate versus long-term impact of choices made. Additionally, delays in emotional processing could lead to relationship misunderstandings in interacting with partners or their families.

Finally, rural Niger is a patriarchal culture in which men lead households[10], and consequently, married adolescent girls and young women (AGYW) have less social standing/capital and power given their gender and ages. When AGYW marry in Niger, they move into the home of their husbands, with great influence on them from their mothers-in-law[9]. Rural Nigerien AGYW have more confined mobility in their first year of marriage, in particular (i.e., they are unable to go to the markets, but are allowed to visit family, see neighbors, and attend some basic ceremonies)[10]. They may also be expected by husbands and family to become pregnant during that first year[10], and embrace the concept themselves, supported by the notion that their primary gender roles in the community are to be wives and mothers[11]. Perlman notes that “A woman’s greatest asset in rural Hausa communities is her fertility.”[10] However, he also mentions that some women do engage in income generation with the permission of their husbands[10]. Data from the 2018 Reaching Married Adolescents study suggests most marriages (99%) among AGYW in Dosso region, Niger, specifically, appear stable and intact over a two-year period (data not shown). Additional evidence in the literature related to stability of early marriages in Niger is limited. Analyzed Demographic and Health Survey data from across Niger among 15-49-year-old women, in the longer term, revealed that 25.0% of first unions had concluded, 4.4% through widowhood and 20.6% through divorce, 15–19 years after formation of the relationship[24].

VYA girls, in particular, may be especially susceptible to their low social standing, the isolation, and the pressure to reproduce. Their new home lives may be a lonely place for them given the young age at which this separation from their former village lives or schooling, occurs. The isolation also has the potential to constrain a VYA girl’s identity formation given the importance that social interactions with peers[25, 26] and those in the community, among others, can play in helping to shape identity, as Fandrem points out[27]. Evidence from other low and middle income countries (LMIC) (India and China), suggests that very young adolescence is a time when gender attitudes are being shaped and reinforced[28] by family and peers[29], whether for the positive or negative. In these more isolated home environments, VYA girls may perceive they have little voice and may embrace beliefs about themselves and gender attitudes that slow their uptake of contraceptive and place them at future risk for unintended pregnancies.

### Call to action, gap in the literature, and study purpose

Global health researchers and development stakeholders have called for increased attention to and research on VYA in the last decade, particularly regarding their sexual and reproductive health (SRH)[30–32]. Woog and Kagestan highlight that >750,000 births occurred globally among adolescent girls under 15 in 2016 and that this segment of the population is expected to grow from 143 million to 193 million in Africa, alone, in 2030[33]. In settings such as Niger where marriage of VYA girls is prevalent[4] and their fertility is high[34], this call takes on heightened urgency. Some of the critical information required for improving the health and safety of married VYA girls in Niger includes evidence related to their contraceptive use, unintended pregnancy, and experiences and effects of gender inequities (e.g., IPV and RC). Importantly, we also need to understand whether and how these may differ from the experiences of married older AGYW.

In this *exploratory* study, we compare and contrast reproductive health, gender equity attitudes, and experiences of intimate partner violence among a representative sample of married VYA girls (aged 13–14 years) with those AGYW aged 15–16 years and 17–19 years in the rural Dosso region of Niger.

## Methods

### Study data and analytic sample formation

The analyzed data were collected as part of baseline measurements for a cluster-randomized controlled trial assessing the impact of the Reaching Married Adolescents (RMA) intervention on uptake of modern family planning among married AGYW and their husbands in Dosso region, Niger (Clinical Trials ID: NCT03226730). The RMA trial was carried out in the Doutchi, Loga, and Dosso districts of rural Niger, with 16 villages per district (four control, 12 intervention) participating in the study. Details of the sampling design and intervention can be found elsewhere.[35]

Adolescent girls and young women and their husbands were eligible to participate in the study if an AGYW was a married 13–19 year old fluent in Zarma or Hausa. She could not be sterilized and could not have plans to move over the subsequent 18 months or travel more than 6 months across the study period. There were 1157 AGYW and 1156 husbands who met eligibility criteria, consented, and participated in the RMA study at baseline (an 88% participation rate). The final n available for baseline analyses were 1072 AGYW and 1080 men after one village leader requested his village’s data be removed (data for 25 AGYW, 25 husbands) and after some surveys were removed because of grave data incompleteness concerns. Baseline data was collected from April—June 2016.

### Data collection and measurement of reproductive health factors, gender equity, partner violence, and parity

Female research assistants collected survey data on tablets from AGYW in private settings. Questions focused on issues related to demographics, fertility, knowledge of and self-efficacy to use FP, current use of modern FP, unintended last pregnancy (UIP), gender role attitudes, lifetime RC, and lifetime IPV, among other topics.

We created the accurate knowledge of modern FP and self-efficacy to use FP variables by asking each AGYW a series of questions and then creating a sum or a score. AGYW were asked 14 questions primarily probing their knowledge about various forms of modern FP (i.e., oral contraceptive pills, intrauterine devices (IUD), etc.). One point was awarded for each correct answer obtained. AGYW with higher summed scores (14 being the highest) were considered to have more accurate knowledge of modern FP. The self-efficacy to use FP variable was made of responses to five questions that assessed an AGYW’s confidence: in her ability to suggest to her husband that they wait a healthy amount of time to have another baby; in her ability to suggest to her husband that that they use a FP method; that she could persuade her husband to let her use a FP method; that she could obtain the FP method she wanted; and that she could use the FP method of her choice, correctly. She was awarded one point, with a maximum of five points, for each question she answered in the affirmative.

Additionally, we constructed variables for current use of modern FP and UIP. Current use of modern FP assessed whether an AGYW was currently using FP and if the form being used was modern (i.e., IUD, injectable, implant, pill, male condom, female condom, emergency contraception, or lactational amenorrhea method). AGYW who were pregnant at the time were excluded. Unintended last pregnancy was constructed from variables asking about whether an AGYW had ever given birth and whether the last birth was mistimed or not wanted. This variable was constructed for all AGYW, versus only those AGYW who had ever had a pregnancy, for greater generalizability.

Gender role attitudes were scored based on a summary measure calculated from responses to three statements to which AGYW were asked to respond either ‘agree’ or ‘disagree’. One point was awarded for a gender equitable response, with a score of 3 representing the most gender equitable views. Statements included 1/There are times when a woman deserves to be beaten, 2/A woman should never question her husband’s decisions even if she disagrees with them, and 3/It is natural and right that men have more power than women in the family.

We also constructed variables for three forms of gender-based violence: lifetime experience of RC, lifetime experience of physical IPV, and lifetime experience of sexual IPV. Lifetime experience of RC was coded as yes if an AGYW indicated that her husband had ever done one or more of the following: made her feel bad or treated her badly for wanting to use a FP method to delay or prevent pregnancy; tried to force or pressure her to become pregnant; took away her FP; kept her from going to the clinic to get FP; said he would leave her if she didn’t get pregnant; or physically hurt her because she did not become pregnant. Lifetime physical IPV was coded as yes if an AGYW answered her husband had ever done one or more of the following to her: pushed, shook, or threw something at her; slapped her; twisted her arm or pulled her hair; hit her with his fist or something that could hurt; kicked, dragged, or beat her up; or choked or burned her. Finally, lifetime sexual IPV was coded as occurring if the participant reported that her husband had ever either physically forced her to have sex when she did not want to, or physically forced her to perform other sexual acts she did not want to. Finally, parity was coded based on whether an AGYW had ever given birth or not. An AGYW was considered nulliparous if she had never given birth (though, she could currently be pregnant), while an AGYW who had given birth to at least one child was considered parous. The total number of births was captured among the parous.

### Analysis

Our analyses were intended to be *exploratory*, with the intent of generating areas for future research, given the small sample of VYA (i.e., 13-14-year-olds; n = 49) in this dataset. We first described baseline characteristics of the married AGYWs and their husbands, by age group (13–14 years, 15–16 years and 17–19 years), highlighting factors related to marriage and parity. We then presented the frequency of reproductive health outcomes, gender role attitudes, and partner violence experienced among the married AGYWs by age group. We compared the proportions for these outcomes in the three age groups, assessing if there was a statistical difference in proportions that existed for 13-14-year-olds as compared to the 15-16-year-olds, and 13-14-year-olds as compared to the 17-19-year-olds, using Pearson’s Chi-Square with a p-value significant at p<0.05. We reported p-values only. When expected cell counts fell below 5, we reported Fisher’s exact p-values with the p-value significant at p<0.05. This same information was also presented stratified by parity status. Median values of continuous variables were used to create cut points for frequency presentation.

We next used logistic regression to calculate odds ratios (ORs) with 95% confidence intervals, exploring the relationship between accurate knowledge of and self-efficacy to use FP, gender equity attitudes, lifetime physical IPV, and lifetime sexual IPV, with current use of modern FP and UIP among 15-16-year-olds and 17-19-year-olds. Owing to the small sample size of the 13-14-year-olds, we did not have adequate statistical power to model these relationships for this age group; however, we chose to present this data for the 15–16 and 17-19-year-olds given the limited sexual and reproductive health data among younger vs. older married AGYW in Niger. We initially explored models stratified by parity status given its important role related to the various exposures and the outcomes, but we did not have sufficient sample size to generate estimates for many outcomes. Instead, unadjusted and adjusted odds ratios (aORs) are presented, controlling for parity status in its continuous form. We did not control for additional confounders given small cell sizes for some exposures. We saw low precision of some estimates owing to these sparse cells. SAS software v9.4[36] was used in all mentioned analyses.

### Ethics

Institutional Review Board approvals for the Reaching Married Adolescents parent study were obtained from the University of California, San Diego (ID: 1604075, 03/08/2016), and the Nigerien Ministry of Health. In line with local norms and independent of a married AGYW’s age, verbal consent for a married AGYW’s participation in the study was given by an AGYW’s head of household and/or husband, and then her informed verbal assent to participate in the study was obtained. Local research partners deemed verbal consent and assent, versus written, most appropriate for engaging participation in the study. We followed World Health Organization Guidelines related to minimizing risk of IPV when both a wife and husband were interviewed. This information is discussed further, elsewhere[35].

## Results

There were 1072 married AGYWs in our analytic sample, with 49 aged 13–14 years, 248 aged 15–16 years, and 775 aged 17–19 years. AGYWs married very young, with the majority of 13-14-year-olds and 15-16-year-olds marrying between the ages of 11 and 14. The mean age difference between husband and AGYW ranged from a low of 8 years among 13-14-year-olds to a high of 9 years among 17-19-year-old AGYWs (see Table 1).

**Table 1:** Baseline characteristics of married AGYWs and their husbands in Dosso region, Niger, by age group (n = 1072).

	13-14-yo n = 49		15-16-yo n = 248		17-19-yo n = 775	
**AGYW**	n (%)		n (%)		n (%)	
Age of AGYW at marriage						
7–10	1 (2.1)		7 (2.8)		22 (2.9)	
11–14	46 (97.9)		157 (63.6)		371 (48.0)	
15 +	0 (0.0)		83 (33.6)		380 (49.2)	
Had first menstrual period	35 (71.4)		236 (95.6)		731 (94.7)	
Ever given birth	6 (12.2)		88 (35.6)		556 (71.8)	
Number of children of AGYW						
0	44 (89.8)		157 (63.6)		224 (28.9)	
1	3 (6.1)		81 (32.8)		289 (37.3)	
2	1 (2.0)		8 (3.2)		198 (25.6)	
3+	1 (2.0)		1 (0.4)		64 (8.3)	
Currently pregnant	4 (8.2)		30 (12.2)		100 (13.0)	
Attended modern school	19 (41.3)		80 (32.4)		273 (35.5)	
**AGYW’s husband**	n (%)	mean (range)	n (%)	mean (range)	n (%)	mean (range)
Age		22 (17, 32)		23 (15, 43)		27 (18, 53)
Age difference between husband and AGYW		8 (3, 18)		8 (-1, 27)		9 (-1, 36)
Number of wives						
1	44 (95.7)		212 (88.7)		641 (84.9)	
2+	2 (4.4)		27 (11.3)		114 (15.1)	
Attended modern school	25 (55.6)		133 (56.1)		344 (45.7)	
In the last 12 months, husband was away more than 3 months from the village	35 (77.8)		168 (70.9)		519 (68.9)	

### Reproductive factors

Current use of modern FP was low, with 4% of 13-14-year-olds as compared to 14% of 17-19-year-olds reporting use (p = 0.07). A higher percent of current use was reported among parous versus nulliparous AGYW (see Table 2 - all AGYW in the sample, Table 3 - parous AGYW, and Table 4 - nulliparous AGYW).

**Table 2:** Frequency of reproductive health, gender equity, and partner violence factors experienced among married AGYWs in Dosso region, Niger, by age group.

	13-14- yo n = 49	15-16- yo n = 248	17-19- yo n = 775	Chi-Square three-way comparison of 13-14-yo vs 15-16-yo vs 17-19-yo*	Chi-Square, two-way comparison of 13-14-yo vs 15-16-yo*	Chi-Square, two-way comparison of 13-14-yo vs 17-19-yo*
	n (%)	n (%)	n (%)			
**Accurate knowledge about FP** score of 3 or higher of 14	15 (30.6)	115 (46.8)	490 (63.6)	< .0001	0.0378	< .0001
**Self-efficacy to use FP** score of 5 out of 5	26 (54.2)	141 (56.9)	520 (67.4)	0.0035	0.7310	0.0601
**Currently use modern FP**†	2 (4.4)	15 (6.9)	93 (13.8)	0.0070	0.7451	0.0719
**UIP**‡	2 (4.1)	34 (13.8)	247 (32.3)	< .0001	0.0582	< .0001
**Personal attitudes related to gender equity** support for more equitable attitudes (score 1 or higher of 3)	24 (50.0)	114 (46.0)	377 (49.1)	0.6762	0.6082	0.9025
**Lifetime experience of RC**	7 (16.7)	25 (11.0)	77 (10.7)	0.4335	0.3025	0.2092
**Lifetime experience of physical IPV**	3 (6.3)	23 (9.4)	62 (8.1)	0.7626	0.7803	1.0000
**Lifetime experience of sexual IPV**	7 (15.2)	17 (6.9)	33 (4.3)	0.0065	0.0772	0.0055

YO = Year-olds; FP = Family planning; UIP = Unintended last pregnancy; RC = Reproductive coercion, IPV = Intimate partner violence

*Pearson’s Chi-Square and degrees of freedom not reported–only p-value reported; Fisher’s exact p-value reported if expected cell size below 5

†Excludes pregnant AGYWs (n = 134)

‡Unintended pregnancy out of all girls and young women in the sample

**Table 3:** Frequency of reproductive health, gender equity, and partner violence factors experienced among married AGYWs in Dosso region, Niger, by age group, *among the parous*.

	13-14- yo n = 6	15-16- yo n = 88	17-19- yo n = 549	Chi-Square three-way comparison of 13-14-yo vs 15-16-yo vs 17-19-yo*	Chi-Square, two-way comparison of 13-14-yos vs 15-16-yos*	Chi-Square, two-way comparison of 13-14-yos vs 17-19-yos*
	n (%)	n (%)	n (%)			
**Accurate knowledge about modern FP** score of 3 or higher of 14	4 (66.7)	63 (71.6)	395 (72.5)	0.8695	1.0000	0.6697
**Self-efficacy to use modern FP** score of 5 out of 5	5 (83.3)	67 (76.1)	400 (73.1)	0.7704	1.0000	1.0000
**Currently use modern FP**†	1 (25.0)	11 (13.6)	93 (18.6)	0.3836	0.4624	0.5625
**UIP**‡	2 (33.3)	33 (37.9)	244 (45.0)	0.3887	1.0000	0.6956
**Personal attitudes related to gender equity** support for more equitable attitudes (score of 1 or higher of 3)	4 (66.7)	34 (38.6)	256 (47.1)	0.2127	0.2174	0.4288
**Lifetime experience of RC**	3 (60.0)	8 (9.3)	56 (10.7)	0.0173	0.0118	0.0113
**Lifetime experience of physical IPV**	0 (0.0)	11 (12.5)	49 (9.0)	0.4723	1.0000	1.0000
**Lifetime experience of sexual IPV**	0 (0.0)	6 (6.8)	22 (4.1)	0.4391	1.0000	1.0000

YO = Year-olds; FP = Family planning; UIP = Unintended last pregnancy; RC = Reproductive coercion, IPV = Intimate partner violence

*Pearson’s Chi-Square and degrees of freedom not reported–only p-value reported; Fisher’s exact p-value reported if expected cell size below 5

†Excludes pregnant AGYWs (n = 56)

‡Unintended pregnancy out of all girls and young women in the sample

**Table 4:** Frequency of reproductive health, gender equity, and partner violence factors experienced among married AGYWs in Dosso region, Niger, by age group, among the nulliparous*.

	13-14- yo n = 43	15-16- yo n = 160	17-19- yo n = 226	Chi-Square three-way comparison, p-value†	Chi-Square, two-way comparison of 13-14-yos vs 15-16-yos†	Chi-Square, two-way comparison of 13-14-yos vs 17-19-yos†
	n (%)	n (%)	n (%)			
**Accurate knowledge about modern FP** score of 3 or higher of 14	11 (25.6)	52 (32.9)	95 (42.2)	0.0459	0.3583	0.0409
**Self-efficacy to use modern FP** score of 5 out of 5	21 (50.0)	74 (46.3)	120 (53.3)	0.3908	0.6648	0.6912
**Currently use modern FP**‡	1 (2.4)	4 (3.0)	0 (0.0)	0.0429	1.0000	0.1934
**UIP**§	0 (0.0)	1 (0.6)	3 (1.3)	0.7674	1.0000	1.0000
**Personal attitudes related to gender equity** support for more equitable attitudes (score of 1 or higher of 3)	20 (47.6)	80 (50.0)	121 (54.0)	0.6244	0.7836	0.4458
**Lifetime experience of RC**	4 (10.8)	17 (12.1)	21 (10.5)	0.9137	1.0000	1.0000
**Lifetime experience of physical IPV**	3 (7.1)	12 (7.6)	13 (5.8)	0.7381	1.0000	0.7237
**Lifetime experience of sexual IPV**	7 (17.5)	11 (7.0)	11 (4.9)	0.0237	0.0602	0.0099

YO = Year-olds; FP = Family planning; UIP = Unintended last pregnancy; RC = Reproductive coercion, IPV = Intimate partner violence

*NB.78 girls who reported being nulliparous (never having given birth) reported being currently pregnant

†Pearson’s Chi-Square and degrees of freedom not reported–only p-value reported; Fisher’s exact p-value reported if expected cell size below 5

‡Excludes pregnant AGYWs (n = 78)

§Unintended pregnancy out of all girls and young women in the sample

Among 17-19-year-olds, the adjusted odds of current use of modern FP among those who reported lifetime experience of physical IPV were 2.11 times that of those who did not report lifetime experience of physical IPV. The estimate for 15-16-year-olds was also raised but was imprecise and not statistically significant (see Table 5 for unadjusted estimates and Table 6 for adjusted estimates).

**Table 5:** Crude associations between reproductive health, gender equity, and partner violence factors with current modern FP use and UIP experienced among married AGYWs in Dosso region, Niger, stratified by age, 15-16-year-olds and 17-19-year-olds.

	Currently use modern FP*	Currently use modern FP*	UIP	UIP
	15-16-yos	17-19-yos	15-16-yos	17-19-yos
	OR, 95% CI	OR, 95% CI	OR, 95% CI	OR, 95% CI
**Accurate knowledge about modern FP** score of > = 3 of 14	CC†	10.18 (4.38, 23.67)	2.76 (1.28, 5.95)	2.10 (1.50, 2.94)
**Self-efficacy to use modern FP** score of 5 out of 5	3.58 (0.98, 13.09)	4.48 (2.27, 8.83)	1.72 (0.80, 3.70)	0.53 (0.39, 0.73)
**Personal attitudes related to gender equity** more equitable attitudes: score of > = 1 of 3	0.40 (0.12, 1.29)	1.01 (0.65, 1.57)	1.20 (0.58, 2.47)	2.27 (1.66, 3.11)
**Lifetime experience of physical IPV**	2.45 (0.63, 9.54)	2.26 (1.18, 4.32)	0.92 (0.26, 3.30)	1.88 (1.11, 3.20)
**Lifetime experience of sexual IPV**	0.88 (0.11, 7.13)	1.59 (0.63, 3.99)	0.81 (0.18, 3.72)	1.28 (0.62, 2.67)

FP = Family planning; IPV = Intimate partner violence; YOS = Year-olds

*Currently use FP excludes those who are currently pregnant (n = 30 for 15-16-year-olds, n = 100 for 17-19-year-olds)

†CC = Cannot compute odds ratio and 95% confidence intervals because of a zero cell.

**Table 6:** The association between reproductive health, gender equity, and partner violence factors with current modern FP use and UIP experienced among married AGYWs in Dosso region, Niger, stratified by age, 15-16-year-olds and 17-19-year-olds, adjusted for parity status*.

	Currently use modern FP†	Currently use modern FP†	UIP	UIP
	15-16-yos	17-19-yos	15-16-yos	17-19-yos
	OR, 95% CI	OR, 95% CI	OR, 95% CI	OR, 95% CI
**Accurate knowledge about modern FP** score of > = 3 of 14	CC†	8.39 (3.58, 19.63)	1.31 (0.55, 3.13)	1.55 (1.08, 2.23)
**Self-efficacy to use modern FP** score of 5 out of 5	2.38 (0.61, 9.27)	3.93 (1.98, 7.80)	0.87 (0.36, 2.07)	0.34 (0.24, 0.49)
**Personal attitudes related to gender equity** more equitable attitudes: score of > = 1 of 3	0.51 (.15, 1.70)	1.08 (0.69, 1.70)	2.11 (0.91, 4.90)	2.92 (2.07, 4.13)
**Lifetime experience of physical IPV**	1.63 (0.37, 7.18)	2.11 (1.08, 4.11)	0.41 (0.09, 1.90)	1.62 (0.92, 2.85)
**Lifetime experience of sexual IPV**	0.78 (0.09, 6.67)	1.58 (0.61, 4.11)	0.65 (0.12, 3.51)	1.20 (0.54, 2.64)

FP = Family planning; IPV = Intimate partner violence; YOS = Year-olds

*Models control for parity modeled continuously

†Currently use modern FP excludes those who are currently pregnant (n = 30 for 15-16-year-olds, n = 100 for 17-19-year-olds)

‡CC = Cannot compute adjusted odds ratio and 95% confidence intervals because of a zero cell

High self-efficacy to use FP (a score of 5/5) was prevalent among all age groups. Accurate knowledge of modern FP was low among the sample, with a median score of 3 out of 14. A higher proportion of 17-19-year-old AGYWs scored 3 or higher as compared to 15-16-year-olds, and 13-14-year-olds. Statistical differences were seen when comparing the 13-14-year-olds to the 15-16-year-olds and to the 17-19-year-olds (Table 2). The highest percent of accurate knowledge of modern FP (score of 3 or higher) was seen among parous AGYWs as compared to nulliparous AGYWs (Table 3 and Table 4). Report of UIP was lowest among the 13-14-year-olds, followed by the 15-16-year-olds, and then the 17-19-year-olds, with a statistical difference seen when comparing the 13-14-year-olds and 17-19-year-olds. Among 17-19-year-olds, the adjusted odds of UIP among those who reported having more gender equitable attitudes, a score of 1 or higher of 3, were 2.92 times that of those who did not report having more gender equitable attitudes. The estimate for 15-16-year-olds was similar, though not as precise and not statistically significant (see Table 5 for unadjusted estimates and Table 6 for adjusted estimates).

### Violence

Between 11–17% of AGYWs in all age groups reported lifetime RC, with 13-14-year-olds reporting the highest percentage. No statistical differences were seen when comparing the youngest to the older groups. Differences were seen when examining lifetime RC among the parous AGYWs, but not the nulliparous (see Table 3 and Table 4). Noting the small n for parous 13-14-year-olds, lifetime experience of RC among 13-14-year-old parous AGYWs was 60%, while it was 9% among parous 15-16-year-olds and 11% among parous 17-19-year-olds.

The highest percent of lifetime sexual IPV was observed among the 13-14-year-olds (15%) as compared to 7% of girls aged 15–16 and 4% of AGYWs aged 17–19. Nulliparous girls aged 13–14 reported the highest percent of lifetime sexual IPV among the three age groups.

## Discussion

In this *exploratory* study, we sought to examine reproductive health, gender equity attitudes, and partner violence experiences of married AGYW aged 13–14 years, 15–16 years, and 17–19 years in Dosso Region, Niger, and to assess whether statistical differences existed in these outcomes when comparing the age groups. We saw the lowest percents of current use of modern FP, accurate knowledge about FP, self-efficacy to use FP, and UIP among the 13-14-year-olds, followed by the 15-16-year-olds, and then the 17-19-year-olds. Marginal or statistically significant differences were seen in comparing 13-14-year-olds versus 17-19-year-olds for all outcomes. Qualitative evidence has demonstrated that after a first birth has occurred, Nigerien women are more likely to consider birth spacing[11]. Women are said to use various approaches for pregnancy prevention, including herbs; writing passages from the Quran in chalk and then dissolving the chalk in water to drink; charms; and other approaches[10]. Evidence suggests that modern contraception use is low among adolescents 18 or younger in Niger (7%)[9, 37], and parous AGYW in our study were more likely to report current use of modern forms of FP than nulliparous AGYW, in line with the above qualitative work. Accurate knowledge of modern FP was also low among our study, supported by evidence from focus groups conducted with urban and rural Nigerien adolescents that also found lower knowledge related to modern FP, broadly[9]. We note that a higher percent of the older AGYW in our study, seemingly, the older parous AGYW, reported more accurate knowledge of FP as compared to those who were younger. Again, this is consistent with the finding that parous AGYW reported a higher percent of current modern FP use than the nulliparous AGYW. Self-efficacy to use FP, slightly higher among the 17-19-year-olds as compared to the 13-14-year-olds, was additionally driven by those who were parous. Finally, the lower percentage of reported UIP among those aged 13–14 as compared to the older AGYW, was likely related to younger, perhaps, nulliparous VYA wanting to have children and not considering a pregnancy unwanted or mistimed, as compared to older AGYW who already have one or more children.

The level of reported lifetime partner-related RC among the overall group of married AGYWs in our study was high (11–17%). Much of the published literature related to RC is from the United States, though evidence from the LMIC context (e.g., Cote de Ivoire and India) similarly demonstrate a high percent of partner-related RC reported by women. In Cote De Ivoire, 18.6% of women 18 years and older reported lifetime partner-related RC[38], while in Uttar Pradesh, India, 12% of women aged 15–49 reported RC from a current husband or the in-laws[39]. More specifically in our data, while we did not see a difference in proportions across the age groups statistically, we saw 13-14-year-olds reporting the highest percent. In particular, though a small n, we saw 13-14-year-old parous adolescent girls reporting a very high percent of lifetime RC, statistically different from what was seen among both groups of parous older AGYW. Reports of lifetime RC among parous 13-14-year-olds may have been higher than that of older parous AGYW as the fertility intentions between older AGYW and their husbands may be better aligned. Older parous AGYW may already have a number of children in comparison to the parous VYA girls given their younger ages, and older parous AGYW may have greater maturity and ability to communicate with their husbands about their desire for the timing of additional children and need for family planning. Research from Kenya and Malawi highlights the importance of partner communication with linked contraceptive use[40, 41]. Additionally, parous VYA girls may have attempted to obtain or use FP but been less able to cover their attempts or hide it in comparison to older parous AGYW. Qualitative evidence from Maradi, Niger suggests some women are using contraception without the knowledge of their husbands and some hide it[10]. Older AGYW may have a greater number of social connections outside of the immediate family to help them with this. Older AGYW may also have greater mobility to access contraception as compared to parous VYA girls given their older ages.

We also observed a greater percent of lifetime sexual IPV reported among VYA girls as compared to AGYW aged 15–16 and 17–19. Specifically, nulliparous 13-14-year-olds reported a higher percent of lifetime sexual IPV as compared to the two older nulliparous groups; a statistical difference was seen between 13-14-year-olds and 17-19-year-olds. While we could not identify any age-specific evidence on sexual IPV among women in Niger, data from a study of ever-married women aged 18–49 in five regions of Niger suggests that approximately 10.8% of women have experienced physical or sexual IPV from a partner at any point in their lives[42]. In our data, we posit that the higher percent of sexual IPV seen among 13-14-year-olds, specifically, may be due to three main reasons. First, the 13-14-year-olds may have reported sex as forced or unwanted sex if they recently married and were not as familiar with the concept of sex given their young ages in contrast to the older AGYW (Personal communication, Dr. Sani Aliou, October 11, 2019). Secondly, VYA girls may have been more likely to report forced or unwanted sex if sex was accompanied by tearing, severe pain, or other physical problems related to their physical immaturity, which may have made them better able to recall the sexual IPV more easily. Third, forced sex or sexual acts may be more common among those girls who are younger and nulliparous because nulliparous VYA girls may just be experiencing menarche[17]. It may be that the very young adolescents’ husbands are eager to impregnate their wives once they are able to conceive, allowing husbands to demonstrate their virility to their families and communities. They may be having sex, including forced sex, more often with their wives as compared to husbands of older nulliparous AGYW, making VYA girls more likely to remember the events and report them.

These current findings are limited by the small number of VYA girls included in this married adolescent sample. Future research should involve collection of quantitative data from larger samples of VYA girls. Further, qualitative data should be collected from AGYW to explore the contexts of RC and sexual IPV, in particular among VYA, and how their experiences differ from that of married older AGYW. Qualitative research with husbands and his family could also provide additional insights on VYA reproductive health and IPV.

Policymakers and public health practitioners must strengthen efforts to delay age at marriage and childbirth in order to protect the physical and emotional well-being of VYA girls. As part of this effort, VYA girls, their parents, (future) husbands, and the community at large, should be provided with information about the poor health outcomes which could occur to AGYW and infants when girls birth children while in adolescence[6–10]. Moreover, investment is needed in accessible, appropriate sexual and reproductive health services for VYA.

There are both strengths and limitations of this study. Strengths include a focus on Nigerien married VYA girls, a population on which there is limited data. Additionally, given our population-based study design, these results are generalizable to the included districts. As discussed above, a major limitation of this work is that our sample size for the VYA girls is small (n = 49), making this analysis exploratory. Furthermore, the cross-sectional nature of these results prohibits us from establishing clear temporality between relationships. Finally, although we treat parity as a possible confounder in one table of the regression analyses, we acknowledge that it could instead be playing a mediating role in some of these exposure-outcome relationships.

## Conclusions

In summary, this study highlights the reported reproductive, gender equity attitude, and partner violence experiences of married, Nigerien AGYW, comparing experiences of VYA against those of older AGYW. We found differences in reproductive factors, as well as lifetime RC and sexual IPV experiences among 13-14-year-olds as compared to older AGYW. VYA girls should be prioritized in future sexual and reproductive health research initiatives, given their unique vulnerabilities as compared to older AGYW, as well as the global calls for better understanding of VYA needs, both supported by the findings of this current study.

## References

[1] (UNFPA) United Nations Population Fund. World Population Dashboard–Niger. Available from: https://www.unfpa.org/data/world-population/NE.

[2] World Food Programme. Country Brief—Niger. https://www.wfp.org/countries/niger

[3] World Bank. The World Bank in Niger. Available from: https://www.worldbank.org/en/country/niger/overview#1.

[4] UNICEF. Child marriage. Data 2017. October 2019. Available from: https://data.unicef.org/topic/child-protection/child-marriage.

[5] Althabe F, Moore JL, Gibbons L, Berrueta M, Goudar SS, Chomba E, et al Adverse maternal and perinatal outcomes in adolescent pregnancies: The Global Network's Maternal Newborn Health Registry study. Reprod Health. 2015;12(Suppl2):S8. 26063350 10.1186/1742-4755-12-S2-S8 PMC4464033

[6] Ganchimeg T, Ota E, Morisaki N, Laopaiboon M, Lumbiganon P, Zhang J, et al Pregnancy and childbirth outcomes among adolescent mothers: a World Health Organization multicountry study. BJOG-Int J Obstet Gy. 2014; 121 (S1): 40–48. 10.1111/1471-0528.12630 24641534

[7] Mombo-Ngoma G, Mackanga JR, Gonzalez R, Ouedraogo S, Kakolwa MA, Manego RZ, et al Young adolescent girls are at high risk for adverse pregnancy outcomes in sub-Saharan Africa: an observational multicountry study. BMJ Open. 2016; 6: e011783 10.1136/bmjopen-2016-011783 27357200 PMC4932321

[8] Kurth F, Belard S, Mombo-Ngoma G, Schuster K, Adegnika AA, Bouyou-Akotet MK, et al Adolescence As A Risk Factor for Adverse Pregnancy Outcome in Central Africa–A Cross-Sectional Study. PLOS One. 12 2010; 5(12): e14367 10.1371/journal.pone.0014367 21188301 PMC3004789

[9] Barroy H, Cortez R, Le Jean N, Wang H. Addressing Adolescent Sexual and Reproductive Health in Niger. Washington, D.C: The World Bank; 1 2016.

[10] Perlman D, Chaibou S. Sahel Resilience Learning (SAREL): Women’s Empowerment and Resilience in Niger: An Ethnographic Study. Washington D.C: United States Agency for International Development; 10 2018 (revised December 2018).

[11] Camber Collective. Niger Family Planning Demand Analysis: Qualitative Research Brief. February 21, 2014. Available from https://static1.squarespace.com/static/55723b6be4b05ed81f077108/t/58c8571d29687fd3a5bf86dd/1489524521084/Niger_Qualitative+Research+Brief_Final.pdf.

[12] Silverman JG, Raj A. Intimate Partner Violence and Reproductive Coercion: Global Barriers to Women’s Reproductive Control. PLOS Med. 2014;11(9):e1001723 10.1371/journal.pmed.1001723 25226396 PMC4165751

[13] Grace KT, Anderson JC. Reproductive Coercion: A Systematic Review. Trauma, Violence, and Abus. 2018;19(4):371–390. 10.1177/1524838016663935 PMC5577387 27535921

[14] Blum RW, Astone NM, Decker MR, Chaundra-Mouli V. A conceptual framework for early adolescence: a platform for research. Int J Adolesc Med Health. 2014; 26 (3):321–331. 10.1515/ijamh-2013-0327 24486726 PMC4476282

[15] Youthpower. Very young adolescents. Available from: https://www.youthpower.org/youthpower-issues/topics/very-young-adolescents.

[16] UNICEF. The State of the World’s Children: Adolescence, the Age of Opportunity. New York, New York: United Nations Children’s Fund; 2 2011.

[17] Sawyer SM, Afifi RA, Bearinger LH, Blakemore SJ, Dick B, Ezeh AC, et al Adolescence: a foundation for future health. Lancet. 2012; 379:1630–1640. 10.1016/S0140-6736(12)60072-5 22538178

[18] Tamminga CA. Images in Neuroscience: Frontal Cortex Function. Am J Psychiat. December 2004;161(12): 2178. 10.1176/appi.ajp.161.12.2178 15569885

[19] Mills KL, Goddings AL, Herting MM, Meuwese R, Blakemore SJ, Crone EA, et al Structural brain development between childhood and adulthood: Convergence across four longitudinal samples. NeuroImage. 2016;141:273–281. 10.1016/j.neuroimage.2016.07.044 27453157 PMC5035135

[20] Johnson SB, Blum RW, Giedd JN. Adolescent Maturity and the Brain: The Promise and Pitfalls of Neuroscience Research in Adolescent Health Policy. J Adolescent Health. September 2009;45(3):216–221. 10.1016/j.jadohealth.2009.05.016 PMC2892678 19699416

[21] Arain M, Haque M, Johal L, Mathur P, Nel W, Rais A, et al Maturation of the adolescent brain. Neuropsych Dis Treat. 2013; 9: 449–461. 10.2147/NDT.S39776 PMC3621648 23579318

[22] Steinberg L, Graham S, O'Brien L, Woolard J, Cauffman E, Banich M. Age Differences in Future Orientation and Delay Discounting. Child Dev. Jan-Feb 2009; 80(1): 28–44. 10.1111/j.1467-8624.2008.01244.x 19236391

[23] McGivern RF, Andersen J, Byrd D, Mutter KL, Reilly J. Cognitive efficiency on a match to sample task decreases at the onset of puberty in children. Brain Cogn. 2002;50:73–89. 10.1016/s0278-2626(02)00012-x 12372353

[24] Clark S, Brauner OS. Divorce in sub-Saharan Africa: Are unions becoming less stable? Popul Dev Rev. 12 2015;41(4): 583–605.

[25] McLean KC, Jennings LE. Teens telling tales: How maternal and peer audiences support narrative identity development. J Adolescence. 2012; 35:1455–1469. 10.1016/j.adolescence.2011.12.005 22209556

[26] Brinhaupt TM, Lipka RP. Chapter 1: Understanding Early Adolescent Self and Identity: An Introduction In: Brinhaupt TM, Lipka RP, editors. Understanding Early Adolescent Self and Identity: Applications and Interventions. Albany, New York: State University of New York Press; 2002. p1-21.

[27] Fandrem H. Friendship During Adolescence and Cultural Variations In: Wright J, Editor: International Encyclopedia of the Social and Behavioral Sciences. 2nd Edition Elsevier; 2015 p432–441.

[28] Basu S, Zuo X, Lou C, Acharya R, Lundgren R. Learning to Be Gendered: Gender Socialization in Early Adolescence Among Urban Poor in Delhi, India, and Shanghai, China. J Adolescent Health. 2017;61(4S):S24–S29. 10.1016/j.jadohealth.2017.03.012 28915988

[29] Kagesten A, Gibbs S, Blum RW, Moreau C, Chandra-Mouli V, Herbert A, et al Understanding Factors that Shape Gender Attitudes in Early Adolescence Globally: A Mixed-Methods Systematic Review. PLOS One. 2016;11(6):e0157805 10.1371/journal.pone.0157805 27341206 PMC4920358

[30] Igras SM, Macieira M, Murphy E, Lundgren R. Investing in very young adolescents’ sexual and reproductive health. Glob Public Health. 2014; 9(5): 555–569. 10.1080/17441692.2014.908230 24824757 PMC4066908

[31] Lane C, Brundage CL, Kreinin T. Why We Must Invest in Early Adolescence: Early Intervention Lasting Impact. J Adolescent Health. 2017; 61: S10–S11. 10.1016/j.jadohealth.2017.07.011 PMC5612021 28915985

[32] Dixon-Meuller R. The sexual and reproductive health of young adolescents in developing countries: Reviewing the evidence, identifying research gaps, and moving the agenda Report of a WHO technical consultation, Geneva, 4–5 November 2010. Geneva, Switzerland: World Health Organization, 2011 Available at https://apps.who.int/iris/bitstream/handle/10665/70569/WHO_RHR_11.11_eng.pdf?sequence=1&isAllowed=y

[33] Woog V, Kagestan A. The Sexual and Reproductive Health Needs of Very Young Adolescents Aged 10–14 in Developing Countries: What Does the Evidence Show? New York: Guttmacher Institute; 2017 Available at https://www.guttmacher.org/sites/default/files/report_pdf/srh-needs-very-young-adolescents-report_0.pdf.

[34] UNICEF. Progress for Children: A report card on adolescents. Number 10. New York, New York: UNICEF, 2012.

[35] Challa S, DeLong SM, Carter N, Johns N, Shakya H, Boyce SC, et al Protocol for cluster randomized evaluation of reaching married adolescents—a gender-synchronized intervention to increase modern contraceptive use among married adolescent girls and young women and their husbands in Niger. Reprod Health. 2019;16:180 10.1186/s12978-019-0841-3 31852538 PMC6921454

[36] SAS Version 9.4. SAS Institute Inc, Cary, NC.

[37] Institut National de la Statistique (INS) et ICF International. Enquête Démographique et de Santé et à Indicateurs Multiples du Niger 2012. Calverton, Maryland, USA: INS et ICF International, 2013.

[38] Falb KL, Annan J, Kpebo D, Gupta J. Reproductive coercion and intimate partner violence among rural women in Cote d'Ivoire: a cross-sectional study. Afr J Reprod Health. 12 2014;18(4):61–69. 25854094 PMC5783178

[39] Silverman JG, Boyce SC, Dehingia N, Rao N, Chandurkar D, Nanda P, et al Reproductive coercion in Uttar Pradesh, India: Prevalence and associations with partner violence and reproductive health. SSM—Population Health. 2019; 9 100484 10.1016/j.ssmph.2019.100484 31998826 PMC6978494

[40] Tumlinson K, Speizer IS, Davis JT, Fotso JC, Kuria P, Archer LH. Partner communication, discordant fertility goals, and contraceptive use in urban Kenya. Afr J Reprod Health. 2013;17(3):79–90. 24069770 PMC3786372

[41] Hartmann M, Gilles K, Shattuck D, Kerner B, Guest G. Changes in couples' communication as a result of a male-involvement family planning intervention. J Health Commun. 2012;17(7):802–819. 10.1080/10810730.2011.650825 22545820

[42] John NA, Edmeades J, Murithi L. Child marriage and psychological well-being in Niger and Ethiopia. BMC Public Health. 2019;19:1029 10.1186/s12889-019-7314-z 31370825 PMC6670185

